# Analysis of Variables Influencing Short-Term Complications in Geriatric Patients Undergoing Emergency General Surgery (EGS): A Retrospective Cohort Study

**DOI:** 10.7759/cureus.44031

**Published:** 2023-08-24

**Authors:** Abubaker Elamin, Laith Sinan, Seyedh Paniz H Tari, Bilal I Ahmad

**Affiliations:** 1 General Surgery, Humanitas University, Milan, ITA; 2 Otolaryngology, Nottingham University Hospitals, Nottingham, GBR; 3 Orthopaedic Surgery, Nottingham University Hospitals, Nottingham, GBR; 4 Orthopaedic Surgery, United Lincolnshire Hospitals, Lincoln, GBR

**Keywords:** blood transfusions, vasopressors, geriatrics, short term complications, : emergency general surgery

## Abstract

Background: Emergency general surgery (EGS) encompasses a wide range of acute surgical conditions that pose significant risks to patient life and well-being. Understanding the factors that contribute to short-term complications in geriatric patients undergoing EGS is crucial for improving patient outcomes. This retrospective single-center cohort study aimed to evaluate the impact of various variables on short-term complications in geriatric patients undergoing EGS.

Methods: A total of 212 patients aged 65 and above who underwent emergency abdominal surgery between 2017 and 2018 were included in the study. The analysis focused on several variables, including age, sex, body mass index (BMI), beta-blocker use, open abdomen treatment, blood transfusions, anticoagulant therapy, and vasopressor use. Univariate and multivariate analyses were conducted to assess the association between these variables and short-term complications.

Results: Among the analyzed variables, blood transfusions and vasopressor use demonstrated a statistically significant association with short-term complications. Patients who received blood transfusions had a significantly higher risk of complications, with an odds ratio (OR) of 3.01 (95% confidence interval, CI: 1.28-7.06, p-value = 0.011). Similarly, the use of vasopressors was strongly correlated with increased short-term complications, with an OR of 14.61 (95% CI: 4.86-43.89, p-value < 0.001).

Conclusion: These findings emphasize the importance of minimizing blood transfusions and careful consideration of vasopressor use in geriatric patients undergoing EGS to reduce the risk of short-term complications. Further research is warranted to explore additional factors and optimize perioperative management strategies to improve outcomes in this vulnerable patient population.

## Introduction

The term emergency general surgery (EGS) has been the subject of much debate among medical professionals in recent years, as a clear and concise definition has yet to be established. In 2013, the American Association of Trauma (AAST) defined EGS as "any patient (inpatient or emergency department) requiring an emergency surgical evaluation (operative or non-operative) for diseases within the realm of general surgery as defined by the American Board of Surgery." Another potential definition is "the urgent assessment and treatment of non-trauma general surgical emergencies involving adults," which is less specific but still captures the overarching themes of EGS.

The EGS encompasses diverse, unique pathologies that carry an acute risk to life or long-term morbidity. For this reason alone, it is a specialty that needs thorough understanding and great emphasis. Although the increased risk of morbidity and mortality has been well established [1], the specific causative factors are poorly understood. However, they are generally attributed to patient-related physiological derangements and pre-existing co-morbidities [2-4].

Incidence

In the United States, there are about 130 million emergency room visits each year, which is a significant number. However, projections indicate that this number will continue to rise in the coming years [5-6]. Out of these 130 million visits, around 27 million are EGS admissions, representing a 28% increase since 2001 [7]. This means there are now more admissions for EGS than for cancer and diabetes combined, highlighting this field's growing importance in healthcare [8].

While geriatric patients make up only 15% of the population in the United States, they account for over 30% of EGS procedures. This is attributed to several factors, including a higher prevalence of co-morbidities and frailty among elderly patients [9]. In the United Kingdom, EGS accounts for about 50% of the surgical workload completed by the National Health Service (NHS) but makes up nearly 80% of surgical deaths due to the high number of complications and reduced time for optimizing pre-surgical risks [10]. In order to better comprehend the risks associated with each surgery, it is essential to understand which variables have the highest impact on patient morbidity. In this way, we can better determine the likelihood of a patient having a specific outcome. Our research aimed to evaluate which variables had the most significant effects on these patient outcomes, particularly regarding short-term complications.

## Materials and methods

In order to find the validity of our hypothesis, we performed a retrospective single-center cohort study. Information was gathered for all patients over 65 who underwent EGS in Humanitas Research Hospital, Milan, Italy. Patient files were accessed through the hospital's encrypted computer system using authorized credentials. These files contained comprehensive information about each patient's admission, including relevant demographics, co-morbidities, and investigations performed such as blood reports and imaging from admission to discharge. 

Inclusion criteria and exclusion criteria

The inclusion criteria were as follows: patients above 65 years of age. Patients who had undergone an emergency abdominal surgery over two years between 2017 and 2018 (24 months). Admission type to the surgical unit was urgent for the pathologies covered throughout [appendicitis, cholecystitis, diverticulitis, peptic ulcer disease (PUD), acute mesenteric ischemia (AMI), and volvulus].

The exclusion criteria were as follows: we excluded patients who were under the age of 65, undergoing elective surgery, having an emergency surgery unrelated to general surgery, trauma patients, patients needing EGS but treated with non-operative management (not fit for surgery), and patients treated with interventional radiology instead of surgery.

Database

The variables included in the database were numerous but can be defined into significant groups. A completed copy of the database is available upon request, it includes: demographic details (patient ID, file number, surname, name, sex, age), dates (admission, intervention, discharge), health parameters [body mass index, BMI, blood pressure, heart rate (initial values were used), ASA], medications [use of beta-blockers, anticoagulants (type), amines (duration and type), or antibiotics], diagnosis (if contrast-enhanced CT was performed, diagnosis, surgery performed, lactate levels), intraoperative factors [type of surgery (laparotomy or laparoscopy), if an open abdomen technique was used after surgery, duration of the surgery, volume of blood loss and any intraoperative blood transfusions, complications experienced intraoperatively and use of a vaccum-assisted closure (VAC) or negative pressure wound therapy (NPWT) system], and postoperative factors [ICU admission and duration, number of days admitted as an inpatient (non-ICU), blood transfusions, use of ventilation (duration and type), reinterventions, medical or surgical complications post-operatively including delirium and complication after discharge].

Data analysis

Our analysis included investigating the necessity for blood transfusions in patients who underwent the 'open abdomen' technique. Additionally, we examined the effect of medications such as beta-blockers, anticoagulants, and amines on patient outcomes. Univariate and multivariate analyses were conducted to assess the impact of these factors on the OR. A p-value of <0.05 was considered to be statistically significant. The statistical program used was 'Stata version 15' (StataCorp. 2017. Stata Statistical Software: Release 15. StataCorp LLC, College Station, TX).

## Results

**Figure 1:**
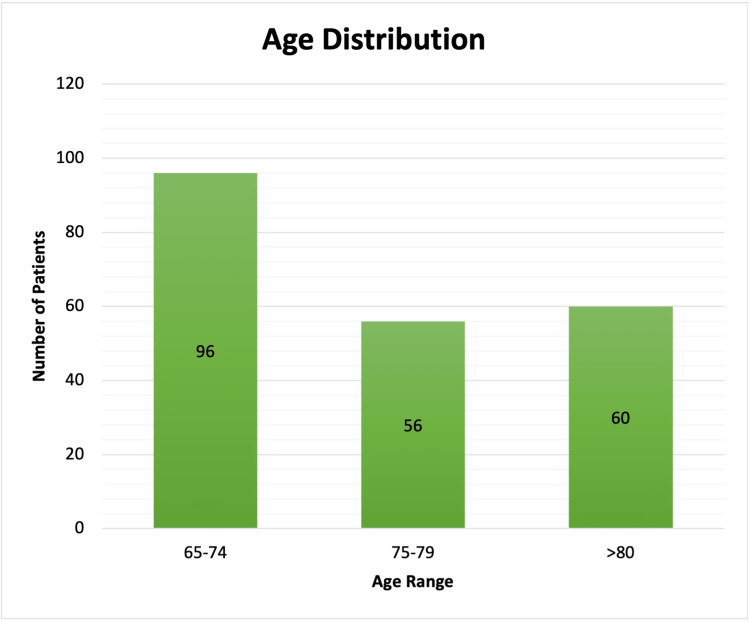
Age distribution.

Age distribution and operations: Two hundred and twelve geriatric patients underwent EGS during our two-year study period. Of these, 96 were aged between 65 and 74, 56 were between 75 and 79, and 60 were above 80 (Figure 1). The surgeries performed included: appendectomies, cholecystectomies, enterotomies and colectomies, abdominal wall surgeries, adhesiolysis, and repair of perforations.

Gender: 116 of the patients were male, whilst the remaining 96 were female, equating to 54.7% and 45.3%, respectively.

**Figure 2:**
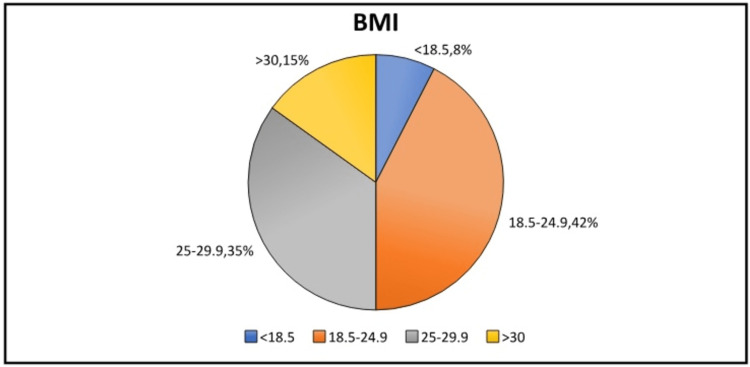
BMI distribution. BMI, body mass index

Body mass index (BMI): Around 7.55% of patients were classified as underweight, 42.45% were normal weight, 34.91% were overweight, and 15.09% were obese, subdivided into Class 1, 2, and 3 depending on severity (Figure 2).

Beta-blockers: Seventy-two patients (34%) were taking beta-blockers for a pre-existing condition, the most commonly used one was Bisoprolol.

Anticoagulants: Of all the patients included, only six were not given an anticoagulant. This meant 97.17% of patients underwent prophylactic low molecular weight heparin (LMWH) therapy during admission.

CT: A pre-operative contrast-enhanced CT scan was performed in 128 patients (60%).

Surgical approach: 118 (56%) of the surgeries were performed laparoscopically, whilst the remaining 94 (44%) were done with an open approach, laparotomy.

Timing: The average duration of surgery was 136 min for all patients, with the range being between 30 and 605 min.

**Figure 3:**
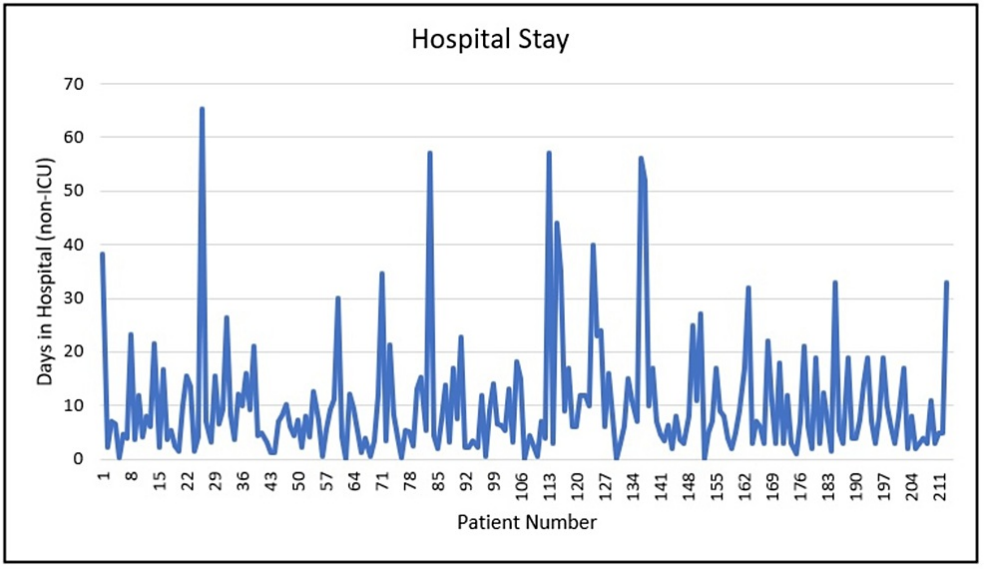
Length of hospital stay.

Duration of stay: The length of stay for patients in the study varied from 0 days for those discharged on the same day as their operation to a maximum of 65 days. On average, all patients stayed in the hospital for 10.26 days (Figure 3). Of the total patient population, 41 out of 212 patients (19.34%) were admitted to the ICU. The average length of stay for patients in the ICU was 22.07 days. 

Medical therapy: Of the 212 patients, all but two were treated with antibiotics. The specific drugs can be found in column 'AS' of the database. The average length of treatment was 10.16 days.

Vasopressors: During their hospital stay, 29 patients required amines (adrenaline, noradrenaline, dobutamine). The minimum duration of use was one day, while the maximum was 11 days.

Transfusion: 52 patients (24.5%) required a blood transfusion during their admission.

Reinterventions: A total of 37 patients (17.5%) had to undergo reinterventions (25 patients had one reintervention, 10 patients had two reinterventions, one patient had three reinterventions, and one final patient had four reinterventions). Of these 37 patients, 20 were treated with the 'open abdomen' approach, meaning these reinterventions were planned procedures. Taking this into account, only 17 patients (8.01%) underwent unplanned reinterventions.

Delirium: Post-surgical delirium was found in nine patients (4.24%).

**Figure 4:**
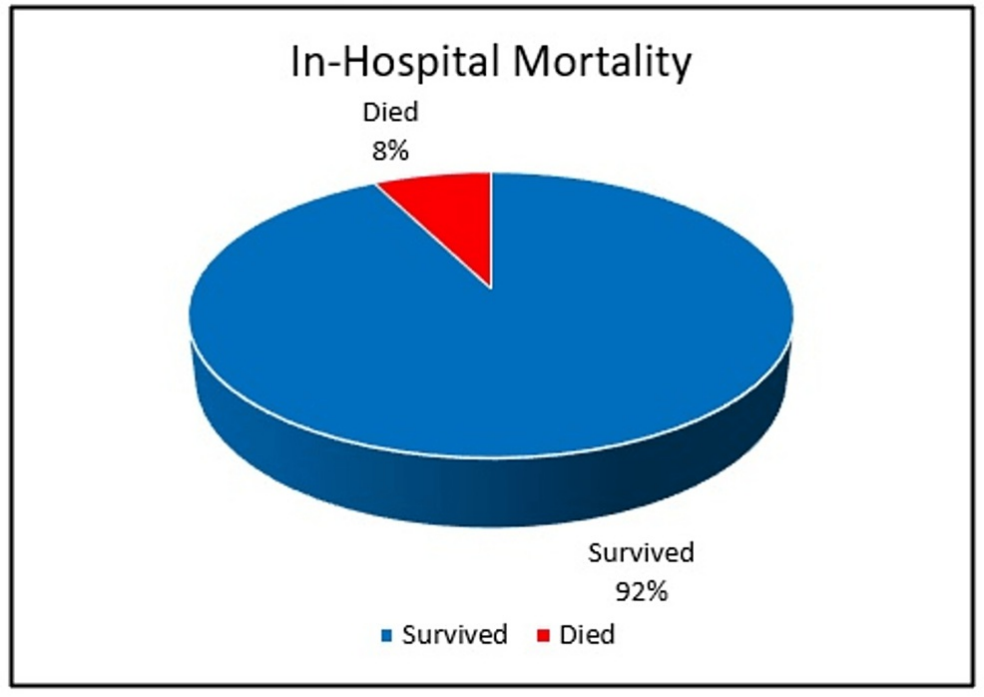
In-hospital mortality.

Mortality rate: Out of the total number of patients included in the study, 7.55% died while admitted, which equates to 16 patients. The 30-day mortality rate, which refers to death occurring within 30 days of surgery, was found to be 6.60%. This figure includes 12 patients who died in the hospital and an additional two who died after being discharged, for a total of 14 patients (Figure 4).

Variables' calculation

We analyzed the variables in the database to objectively analyze their correlation with short-term complications. Both univariate studies and multivariate studies were performed in order to see if other variables influenced the correlation. Once again, we calculated these variables regarding their effect on short-term complications (Table 1).

**Table 1:** Short-term complications associated with variables.

	Univariate		Multivariate	
Variables	OR (95% CI)	p Value	OR (95% CI)	p Value
Age	1.02 (0.97-1.08)	0.354		
Sex	0.88 (0.45-1.73)	0.711		
BMI	1.01 (0.94-1.08)	0.750		
Time to surgery	1.001 (0.998-1.004)	0.467		
Beta-blockers	2.35 (1.18-4.68)	0.015	1.48 (0.64-3.39)	0.357
Open abdomen	5.71 (2.23-14.60)	<0.001	2.37 (0.70-7.99)	0.165
Blood transfusion	5.93 (2.86-12.26)	<0.001	3.01 (1.28-7.06)	0.011
Anticoagulants	0.11 (0.02-0.64)	0.013	0.39 (0.037-4.07)	0.430
Amines	22.72 (8.22-62.76)	<0.001	14.61 (4.86-43.89)	<0.001

Short-term complications

Univariate and multivariate studies were performed for short-term complications. All the OR recorded were within their 95% CI. The male sex had an OR of less than one (0.88), indicating a positive correlation. Anticoagulants had an OR of 0.11, which positively correlated with decreased short-term complications. In the univariate analysis, beta-blockers, open abdomen, blood transfusion, anticoagulants, and amines had a statistically significant p-value of less than 0.05. However, a subsequent multivariate analysis revealed that only blood transfusions and amines remained significant, with a p-value less than 0.05. During our analysis, we decided to include nine unrelated criteria that had the potential for the most effect on the outcome. These are shown in Table 1 and include; Age, Sex, BMI, Time to surgery, Beta-blockers, Open abdomen treatment, Blood transfusions, Anticoagulants, and Amines. Univariate studies were performed for all the variables, and if a significant p-value was obtained, then a multivariate study was performed to determine how significant this value was. Once again, these tests were performed regarding short-term complications.

Short-term complications: multivariate analysis

Male sex: This was the only variable with a negative OR concerning its effect on short-term complications. This means it can be considered a protective factor in outcomes for patients undergoing EGS. We obtained an OR of 0.88 with a 95% CI of 0.45-1.73. The p-value was determined to be 0.711, which translates to statistically insignificant (Table 1).

Age, BMI, and time to surgery: All of these had a positive OR above one and were within their respective CI meaning they were valid results. However, their p-values were above 0.05 and, therefore, statistically insignificant.

Beta-blockers: Upon initial investigation using the univariate test, the OR was calculated to be 2.35 with a CI of 1.18-4.68 and a p-value of 0.015. However, in the multivariate tests, the OR was 1.48 with a CI of 0.64-3.39 and a non-significant p-value of 0.357. These results suggest that other factors may significantly impact complication rates more than beta-blockers, as shown in Table 1.

Anticoagulants: Although during univariate tests they had a p-value of 0.013, this was increased to 0.430 on multivariate testing, meaning that, similar to beta-blockers, their supposedly positive effect was actually due to other factors (Table 1).

Open abdomen treatment: For the univariate study, we found an OR of 5.71 with a 95% CI of 2.23-14.60. The p-value was statistically significant, with a value of <0.001. Upon further examination with a multivariate test, the OR became 2.37 with a CI of 0.70-7.99, and the p-value rose to 0.165, equating to a statistically insignificant difference (Table 1).

Blood transfusions: Once again, blood transfusions produced statistically significant results. This means they are a great predictor of complications in the patients being operated on. The OR during the multivariate analysis was 3.01, with a CI of 1.28-7.06 and a p-value of 0.011 (Table 1). As the p-value remained below 0.05 for both studies, we can confidently assume it is an excellent predictor.

Vasopressors: As before, these drugs remained the most significant factor. Upon multivariate analysis, we found a broad CI of 4.86-43.89 and an OR of 14.61. Although the CI is wide, it is still a significant result, especially when the p-value is considered, which remained <0.001 (Table 1). Overall we can definitively state that vasopressors have the best prediction index for short-term complications.

## Discussion

This article aimed to determine which variables significantly affected outcomes, focusing on short-term complications as our end goal. In the database, over 30 variables were included ranging from demographic criteria such as age or sex to more specific variables, for example, treatment with a VAC system. Short-term complication analyses of these nine criteria (Table 1) revealed that only two factors had a significant effect. These were blood transfusions and vasopressors with p-values of 0.011 and <0.001 respectively. This meant transfusions and vasopressors were the best predictors of morbidity.

In terms of gender, the OR for male sex was less than 1 (0.8) hence suggesting a potential protective factor. The 95% CI of 0.45-1.73 highlights the uncertainty surrounding the association between male sex and short-term complications. The p-value associated with the 'male' sex was determined to be 0.711, which indicates a lack of statistical significance. While the statistical analysis did not demonstrate a significant association between male sex and short-term complications, it is important to consider the potential biological and physiological differences between males and females that could contribute to variations in surgical outcomes. Therefore, further research is warranted to explore the potential mechanisms underlying the observed trend and to better understand the relationship between gender and surgical outcomes in the geriatric population. For this, a larger sample size should be studied.

Interestingly, we found that BMI was not a significant predictor of short-term complications in our study. This contradicts previous studies that identified obesity as a risk factor for postoperative complications in general surgery [11]. However, it should be noted that our study only included a small proportion of obese patients (15.09%), which may have contributed to this finding. In addition, we found that beta-blocker usage was relatively common in our population, with 34% of patients taking it for pre-existing conditions. This underscores the importance of medication management in the perioperative period, as certain medications may affect patient outcomes. Several factors may explain why beta-blocker use lost statistical significance in the multivariate analysis. Firstly, the influence of other variables in the model, such as patient comorbidities, perioperative management strategies, and surgical complexity, could have overshadowed the impact of beta-blockers on short-term complications. These factors might have a more direct and substantial influence on patient outcomes, making the effect of beta-blockers relatively less prominent in this specific population.

Our analysis revealed that a significant proportion of our patients (97.17%) received prophylactic LMWH anticoagulant therapy while being hospitalized. This emphasizes the crucial role of preventing thromboembolic events in this particular group.

We also found that laparoscopic surgery was the preferred surgical approach in over half of the cases (56%), indicating the potential benefits of minimally invasive techniques in this population. Due to continuous improvements in this novel therapy, much has been done to limit the rates of short-term complications. According to our study, these techniques have worked as we found no significant correlation between the available treatment and increased complication rates.

The results demonstrate the significant impact of both blood transfusions and vasopressors on short-term complications in the geriatric population undergoing EGS. These results highlight the importance of considering these variables when assessing the risk of complications in this specific patient group. Blood transfusions emerged as a significant predictor of complications in the study population. The obtained OR of 3.01 suggests that patients who received blood transfusions were three times more likely to experience short-term complications compared to those who did not. The statistical significance of this result, indicated by a p-value of 0.011, further supports the significance of this finding. The CI of 1.28-7.06 indicates a relatively narrow range, implying a higher degree of precision in estimating the association between blood transfusions and complications. These findings are consistent with previous studies that have linked blood transfusions to increased risks of adverse outcomes, such as infections, organ dysfunction, and immune reactions [12].

The administration of vasopressors was identified as the most significant factor associated with short-term complications. The obtained OR of 14.61 highlights a substantial increase in the likelihood of complications in patients receiving vasopressors. Although the CI of 4.86-43.89 is relatively wide, it still encompasses a considerable effect size. The highly significant p-value (<0.001) further confirms the strong association between vasopressor use and the occurrence of complications. Vasopressors are commonly administered to maintain blood pressure and perfusion during EGS. However, their vasoconstrictive properties can lead to complications, including impaired tissue perfusion and subsequent organ dysfunction [13]. Given the significant risk associated with vasopressor use, careful monitoring and individualized administration regimens should be implemented to reduce complications in geriatric patients undergoing EGS. Overall, future research should focus on exploring alternative methods to reduce these risks and enhance perioperative care for geriatric patients undergoing EGS.

Our study provides valuable insights into the factors that may impact short-term complications in geriatric patients undergoing EGS. However, several limitations should be considered. Firstly, our study was retrospective, limiting our ability to establish causality. Secondly, our study was conducted at a single center, which may limit the generalizability of our findings to other populations. The study did not include a control group which could limit the ability to draw causal inferences from the results. Despite these limitations, our study highlights the importance of considering a range of variables in managing geriatric patients undergoing EGS. Future studies could build on our findings by examining additional factors, such as the impact of frailty and cognitive impairment on patient outcomes.

## Conclusions

In conclusion, our study aimed to identify which variables significantly affected short-term complications in geriatric patients undergoing EGS. The study provides valuable insights into the variables that impact short-term complications in geriatric patients. Blood transfusion and vasopressor use were found to have the most significant effect on outcomes. By identifying these factors, healthcare providers can take steps to optimize patient care and improve outcomes in this vulnerable population.

## References

[1] Akinbami F, Askari R, Steinberg J (2011). Factors affecting morbidity in emergency general surgery. Am J Surg.

[2] Havens JM, Peetz AB, Do WS (2015). The excess morbidity and mortality of emergency general surgery. J Trauma Acute Care Surg.

[3] Shafi S, Aboutanos MB, Agarwal S Jr (2013). Emergency general surgery: definition and estimated burden of disease. J Trauma Acute Care Surg.

[4] Ball CG, Hameed SM, Brenneman FD (2010). Acute care surgery: a new strategy for the general surgery patients left behind. Can J Surg.

[5] Villet R (1991). [Overspecialization in surgery]. Chirurgie.

[6] Gale SC, Shafi S, Dombrovskiy VY (2014). The public health burden of emergency general surgery in the United States: a 10-year analysis of the nationwide inpatient sample--2001 to 2010. J Trauma Acute Care Surg.

[7] Garner JP, Prytherch D, Senapati AO (2006). Sub-specialization in general surgery: the problem of providing a safe emergency general surgical service. Colorectal Dis.

[8] Havens JM, Neiman PU, Campbell BL (2019). The future of emergency general surgery. Ann Surg.

[9] Aucoin S, McIsaac DI (2019). Emergency general surgery in older adults: a review. Anesthesiol Clin.

[10] Hsia RY, Kellermann AL, Shen YC (2011). Factors associated with closures of emergency departments in the United States. JAMA.

[11] Kassahun WT, Mehdorn M, Babel J (2022). The impact of obesity on surgical outcomes in patients undergoing emergency laparotomy for high-risk abdominal emergencies. BMC Surg.

[12] Ferraris VA, Hochstetler M, Martin JT (2015). Blood transfusion and adverse surgical outcomes: the good and the bad. Surgery.

[13] Bangash MN, Kong ML, Pearse RM (2012). Use of inotropes and vasopressor agents in critically ill patients. Br J Pharmacol.

